# Augmented Reality in Educational Inclusion. A Systematic Review on the Last Decade

**DOI:** 10.3389/fpsyg.2019.01835

**Published:** 2019-08-13

**Authors:** Jairo Quintero, Silvia Baldiris, Rainer Rubira, Jhoni Cerón, Gloria Velez

**Affiliations:** ^1^GIDATI Research Group, Pontifical Bolivarian University, Medellín, Colombia; ^2^Escuela Superior de Ingeniería y Tecnología (ESIT), International University of La Rioja, Logroño, Spain; ^3^Department of Communication Sciences and Sociology, King Juan Carlos University, Madrid, Spain

**Keywords:** augmented reality, educational inclusion, systematic review, educational technology, disability

## Abstract

The use of Augmented Reality (AR) to achieve educational inclusion has been not deeply explored. This systematic review describes the current state of using AR as an educational technology that takes into consideration the needs of all students including those with a disability. It is done through the analysis of factors, such as the advantages of AR, its limitations, uses, challenges, its scope in the educational field, the attended population and the positive or negative effects of its use in learning scenarios that involve students with diverse educational needs. A total of 50 studies between 2008 and 2018 were analyzed through searching in three interdisciplinary databases: Scopus, Web of Science, and Springer link. For this, the methodological stages considered were planning the review, search, analysis of literature and results report. After analyzing the results, it was possible to demonstrate that the use of AR for inclusive education in the field of sciences is where more studies have been conducted. In regard to the population with disabilities, among the most representative advantages reported were the motivation, interaction and generating interest on the part of the student. At the same time, an important methodological limitation identified was the size of the sample; some investigations were done with two or three subjects, some studies Single Subject Designs were found. In terms of the population attended, the studies generally included students with different impairments (hearing, visual, motor or cognitive), minorities (ethnic, vulnerable), leaving aside other groups excluded as exceptional talents and immigrants, which could be explored in the future. Despite different problems to be addressed, few frameworks to the diversity attention in education were reported, and there was no model and methodology in inclusive education considered in the studies. Finally, from this review we have identified open issues that could give rise to new research in the subject of using AR to favor the creation of inclusive learning scenarios.

## Introduction

According to UNESCO (2017) : “the 2030 agenda for sustainable development, focused on ensuring that no one is left behind, provides a unique opportunity to create more inclusive and equitable societies. This should start with inclusive education systems” (p. 4).

In this regard, for more than two decades, the member countries of UNESCO have implemented policies of educational inclusion, in order to reduce the marginalization and exclusion of students with different learning preferences in the education system, accepting the calls for offering an education that is lifelong and that fits the needs of students (Lindsay, 2018).

The processes of attending the diverse needs of individuals in regards to education, have evolved throughout history from a moralistic viewpoint (Quiroga, 2010), followed by the concept of special education, such as that directed to subjects who, because of various issues: physical, psychological or emotional could not be adapted to traditional teaching (Araque and Barrio, 2010), until now, when they have reached the concept of inclusive education, which is concerned with the right of each child to receive an education according to their individual learning needs (Lindsay, 2018).

The advancement of information and communication technologies (ICT) has not only increased educational coverage by e-learning through the use of a variety of online platforms available today (Lin et al., 2015), but also offers diverse learning experiences that have been shown to impact learning processes in different ways. Proof of this is ubiquitous learning (u-learning), AR (AR), virtual reality (VR), mobile learning (m-learning), games, gamification, or learning analytics (Nincarean et al., 2013). Likewise, recent technological developments have led to mobile devices being used more frequently in education, mainly for children with disabilities or diverse educational needs (Lin et al., 2016a).

Specifically, AR allows combining and superimposing real objects with information and with virtual objects (Azuma et al., 2011). At the same time, the augmented information may not be limited to the sense of sight but may also be applied to all senses, such as hearing, smell and touch (Azuma et al., 2001). This makes AR a promising strategy to favor processes of educational inclusion (Sheehy et al., 2014) since it favors multiple means of representation, of action and multiple ways of engaging students in the learning process (Meyer et al., 2014). There is some scientific evidence to support this claim. For example, Hrishikesh and Nair (2016), in their studies, indicate that AR makes possible for children with disabilities to understand concepts faster and better. Additionally, Mohd Yusof et al. (2014) have shown that AR offers exciting and fun teaching aids for students with special needs as it catches their attention.

Likewise, there is evidence that shows that AR positively impacts the educational experience of students, increases confidence, increases the level of commitment and interest (Fombona et al., 2017), provides opportunities for self-learning (Akçayir and Akçayir, 2017), enhances collaborative learning (Phon et al., 2014), improves satisfaction and increases motivation in students (Liu and Chu, 2010; Di Serio et al., 2013; Bacca et al., 2018).

Literature reviews on the use of AR in education analyze its development, as well as relevant aspects of use, however, as indicated by Gavilanes et al. (2018) when summarizing literature reviews on the use of AR in education until 2017, it is necessary to also analyze the potential of AR to support students with diverse needs, including those with disabilities.

This study presents a systematic review of literature, in which 50 studies that report a direct impact in attending students' diverse needs have been analyzed. The search source of the studies included in the review are databases: Web of Science, Scopus and Springer link. Different keywords were used in the searches in the three databases, the results were crossed to discard repeated documents, obtaining those that met the criteria of the review and following the steps described in the method used.

This document is organized as follows. The first section presents the works related to the present study, those that include AR in education and inclusion. The second section presents the information search process. The third section details the analysis of the literature found. The fourth section describes the results report and finally the fifth section presents the conclusions, challenges and future work.

## Theoretical Framework

This section presents an analysis of existing studies in literature that address the use of AR for the creation of inclusive learning scenarios.

The study reported by Almutairi and Al-Megren (2017) describes the creation of an application with AR to teach Arabic to deaf primary school children, using the potential in combining video, images and audio with AR. The results of the research show that teachers and parents of deaf children prefer using multiple resources; Abas and Zaman (2011) made a storybook to motivate students toward reading. The book was aimed at students who had not reached basic reading skills and were in a recovery period. It consisted of three levels: easy, intermediate and advanced, and managed to provide greater motivation, commitment and a pleasant experience in the immersion of the educational process.

In the studies of Mirzaei et al. (2014) AR was combined with audio and video (AVSR Audio Visual Speech Recognition) in aiding deaf individuals. Through speech recognition techniques, facial expressions allowed capturing what the narrator said, without the need of knowing sign language. By just the use of a screen, the speech became readable text displayed with AR, allowing deaf people to read and better understand what was communicated.

Kerdvibulvech (2016) conducted an AR application study on children with hearing impairment and the goal of the research was helping them communicate both visually and receptively. It applied a portable communication jacket that is linked, called “T.Jacket,” it used sensor technology with AR, and it also extended its application to people with disabilities for its ease in expressing emotions. The result of this evaluation confirmed improvement in understanding and communication.

In contexts of professional training, Bacca et al. (2015) introduced the application called “Paint-cAR,” for students with diverse educational needs, especially for those students with low levels of basic skills and low motivation. The application supports the learning process of re-painting a car, in a vocational education program. This process facilitated students to follow long procedures, which due to their lower level of logical competences and process follow-up, were difficult for them to perform.

In their work, Tobar-Muñoz et al. (2014) designed a digital game with AR called Gremlings [sic] in my Mirror, focused on the development of logical skills in mathematics, which was evaluated with children with diverse learning needs such as: Attention Deficit Hyperactivity Disorder, Autism, Down Syndrome, among others.

The literature reviews on the use of AR in education reported so far have had various objectives. The review carried out by Bacca et al. (2014), as well as that of Chen et al. (2017) highlight the analysis of the current state, reviewing the trends and uses of AR in education, likewise its advantages, limitations and effectiveness. On the other hand, Phon et al. (2014), conducted a review on the use of AR and its potential in educational contexts focused on collaborative learning. Santos et al. (2016) reviewed learning experiences with AR to analyze its usefulness at primary and secondary levels. Espinosa (2015), in her work, focused on projects about education with AR that had been carried out in Spain, as a state of art for that country. Diegmann et al. (2015) reported a systematic review on AR with five areas: discovery-based learning, skills training, training applications, games, and AR books in order to determine their benefits. Fombona et al. (2017) published a synthesis on the relationship between AR and m-learning. In another contribution, Akçayir and Akçayir (2017) presented the systematic review of using AR in educational contexts of formal and informal learning, as well as in trainings in the workplace. Ibáñez and Delgado-kloos (2018) presented a qualitative content analysis between 2010 and 2017 on the use of AR technology to support science, technology, engineering and mathematics (STEM) in learning.

The works reported in the literature reviews describe in detail the current state of use of AR in education, making interesting contributions in regard to the trends and challenges of this area of research. However, none of them reported the current state of knowledge about the use of the AR in education when it comes to aiding the processes of attention to students' diverse needs and for promoting a true educational inclusion. In this context, the research question that guides this study is: What is the current state of use of AR in education in order to support the creation of inclusive learning scenarios?

## Methods

### General Guidelines

In order to carry out the review of the literature object to this study, we considered the guidelines and steps proposed by Egger et al. (2001) in their book Systematic Reviews in Health Care, as well as those of Kitchenham (2004). Specifically, the steps followed for the development of the literature review were the following:

- Planning the review
  1. Ask the question and sub-questions of the review
  2. Definition of preliminary categories of analysis
- Search
  3. Define the sources of literature search
  4. Define the inclusion and exclusion criteria of the literature
  5. Define the search criteria
  6. Search of literature
  7. Selection of literature
- Analysis of literature
  8. Reading of the selected literature
  9. Data extraction and coding
- Results report
  10. Interpretation of results
  11. Generation of the review report

With regard to the analysis of the literature, the recommendations of the Prisma declaration (for reports of systematic reviews and Meta-Analyzes) were followed (Urrútia and Bonfill, 2010; Moher et al., 2015). The PRISMA statement is the international updated version of the QUORUM statement (Quality of meta-analysis reports). In the following sections, each of the steps followed for the review is described in detail.

### Planning the Review

The main research question that this literature review addresses is:

What is the current state of use of AR in education in term of population, interventions, comparators, outcomes and study designs, considering studies between 2008 and 2018 included in three interdisciplinary databases: Scopus, Web of Science and Springer link, in order to support the creation of inclusive learning scenarios?

According to this main research question, a series of research sub-questions were defined:

RQ1: What are the advantages, limitations, effectiveness, uses, challenges, and scope of AR in inclusive education?
RQ2: What are the different types of AR that are the most promising in creating inclusion and why?
RQ3: What types of research designs have been considered when evaluating the use of AR in inclusive education processes?
RQ4: What types of population have been included in the learning scenarios supported by AR?
RQ5: What frameworks or models for attention to diversity have been used to support the creation of AR applications that facilitate processes of educational inclusion?
RQ6: What types of technology, including assistive ones, have been developed to favor the use of AR for educational inclusion?
RQ7: What author's platforms and tools consider the diverse needs of users in the process of creating learning experiences with AR?
RQ8: What is the effect of the AR experiences in terms of outcomes identified in this literature review?

Once the research questions were defined, preliminary analysis categories were established for each sub-question, which could be revisited during the execution of the review. Next, the defined categories are shown.

RQ1: What are the advantages, limitations, uses, challenges and scope of AR in inclusive education?
  - Field of education: based on the International Standard Classification of education (UNESCO, 2013).
  - Reported benefits of AR in inclusive education
  - Reported limitations of AR in inclusive education
  - What are the reported uses of AR in inclusive education?
  - Reported challenges of AR in inclusive education
  - Reported AR achievements in inclusive education
RQ2: What are the different types of AR that are the most promising to favor inclusion and why?
  - Types of AR for inclusion
  - Reasons to be used in the inclusion
RQ3: What types of research designs have been considered when evaluating the use of AR in inclusive education processes?
  - Research method
  - Sample
  - Method of data collection
RQ4: What types of population have been included in the learning scenarios supported by AR?
  - Types of groups with disabilities
  - Types of groups excluded from society
  - Purpose in the diverse population served
RQ5: What frameworks or models for attention to diversity have been used to support the creation of AR applications that facilitate processes of educational inclusion?
  - Working frameworks developed with AR and educational inclusion
  - Models with AR for educational inclusion
  - AR applications in educational inclusion
RQ6: What types of technology, including assistive ones, have been developed to favor the use of AR for educational inclusion?
  - Reported technologies for educational inclusion
RQ7: What platforms and authoring tools consider the diverse needs of users in the process of creating AR-based learning experiences?
  - SDK‘s
  - Programming languages
  - Authoring tools.
  - Software for 3D modeling
  - Other software
RQ8: What is the effect of the AR experiences identified in this literature review?
  - Effect generated in inclusive education

### Search

As research sources, three (3) multidisciplinary databases were selected and recognized for their coverage and indexing, they were consulted and, subsequently, the results were cross-checked: Scopus, SpringerLink, and Web of Science. Scopus is “the largest database of citations and abstracts of refereed literature and high-quality sources on the web” (Andalia et al., 2010), it covers scientific literature that is reviewed by experts, as well as Web of Science; these two are nowadays important sources of consultation (Mongeon and Paul-hus, 2016). On the other hand, SpringerLink is also multidisciplinary, it offers access to more than 8.5 million documents.

#### Criteria for Literature Inclusion

General criteria:

1. Studies published between 2008 and 2018.
2. Studies that describe applications, models or education frameworks for diversity with AR.

Specific criteria in connection to research questions:

- SC.1) Studies that report advantages, limitations, effectiveness, uses, challenges, and the scope of AR in inclusive education.
- SC.2) Studies that describe which are the most promising types of AR to favor inclusion.
- SC.3) Studies that demonstrate the methods of educational evaluation that have been considered for applications of AR in inclusive education.
- SC.4) Studies that contain the types of research designs that they have considered to evaluate the use of AR in inclusive education processes.
- SC.5) Studies that indicate the types of population that have been included in the learning scenarios supported by AR.
- SC.6) Studies that describe frameworks, models or applications of AR that have been developed to support the processes of educational inclusion.
- SC.7) Studies that report what types of technology, including assistive, have been developed to favor the use of AR for educational inclusion.
- SC.8) Studies that indicate the effect of the AR experience.

#### Criteria for Literature Exclusion

The following exclusion criteria were defined and, therefore, the studies that had these issues were discarded:

- Studies or publications that didn't mention the term “AR.”
- Studies that claim to refer to AR but refer to mixed reality or virtual reality.
- Studies of AR that are not oriented in contexts of education for diversity or inclusive education.
- Studies that are not identified as articles, book chapters or conference articles, in the context of AR education and inclusive education.

#### Final Search Criteria

In order to start and have greater clarity, a preliminary search of documents was done, some results were analyzed and it was possible to verify that many studies between 2008 and 2018 have been published on different topics such as: diversity, inclusive education, special education, disability, and universal access in contexts of education with AR. All these were used as keywords and similar results arose when consulting the terms in Thesaurus of UNESCO. Therefore, the following query string was established for each term: “(including AND education and with AND augmented AND reality) AND PUBYEAR > 2007.” A total of six queries were made for each database, changing the keywords for each search and collecting the results.

### Analysis of Literature

Performing the search according to the defined criteria, 363 documents were initially identified among scientific articles, book chapters and conference articles; the first search was made on April 22nd, 2018 and the last on May 3rd of the same year.

A first filter was applied to these 363 studies, evaluating the inclusion and exclusion criteria by considering the title and abstract of each literature, and cross-checking the results of the three databases to discard repeated documents. After this filter, 96 documents remained. Finally, when reading each of the 96 articles, a total of 50 studies met the criteria proposed and defined for the review: 26 journal articles, four book chapters and 20 conferences (Tables 1, 2).

**Table 1:** AR studies in journals.

**Journal/Year of publication**	**Studies**
IEEE Explore: 2010, 2014, 2015	3
Journal of Research on Technology in Education: 2015	2
Journal of Special Education Technology: 2016, 2017	2
Agris on-line Papers in Economics and Informatics: 2015	1
Asia-Pacific Edu Res: 2018	1
Association for Educational Communications & Technology: 2016	1
Educational Technology & Society: 2014	1
Displays: 2015	1
Eurasia Journal of Mathematics Science and Technology Education: 2017	1
International Journal of Heritage Studies: 2018	1
International Journal of Inclusive Education: 2014	1
J Sci Educ Technol: 2015	1
JMIR Human Factors: 2018	1
Journal of Physics: 2016	1
Multimedia Tools and Applications: 2013	1
Procedures Engineering: 2012	1
Research in Developmental Disabilities: 2015	1
Brazilian Magazine (Spanish Edition): 2017	1
Latin Magazine of communication: 2017	1
UCLM Magazine: 2016	1
The Visual Computer: 2013	1
Research in Developmental Disabilities: 2013	1
Total	26

**Table 2:** AR studies in conferences.

**Conferences/Year of publication**	**Studies**
UAHCI–Universal Access in Human-Computer Interaction: 2015, 2016, 2017	3
IVIC–International Visual Informatics Conference: 2011, 2014, 2017	3
CTS–International Conference on Collaboration Technologies and Systems: 2013	1
SVR−15th Symposium on Virtual and AR: 2013	1
ICTE–International Conference on Technology for Education: 2015	1
ICACCI–International Conference on Advances in Computing, Communications and Informatics: 2016	1
IMCL–Interactive Mobile Communication, Technologies and Learning: 2017	1
ICCSA–International Conference on Computational Science and its Applications: 2016	1
IWAAL–International Workshop on Ambient Assisted Living: 2015	1
UCAmI–Ubiquitous Computing and Ambient Intelligence: 2014	1
CCSA–Computational Science and Its Applications: 2016	1
IISA−6th International Conference on Information, Intelligent, Systems and Applications: 2016	1
CHI–Conference on Human Factors in Computing Systems: 2018	1
ICSCC–International Conference on Smart Computing and Communications: 2017	1
VARE–International Conference on Virtual and AR in Education: 2015	1
CIVE 17–V International Conference on Video Games and Education: 2017	1
Total	20

Once the literature to be reviewed was defined, it was read again in detail and the process of extracting and coding the data began, by taking into account the respective preset format for data systematization.

## Results Report

This section describes the results obtained from coding, considering the categories and subcategories established in the planning section of the review with respect to each research sub-question. The list of categories is made according to the research questions (RQ).

Next the findings according to each research question are shown.

- F1: Advantages, limitations, uses, challenges, and scope of AR in inclusive education.

The main advantages reported in the studies analyzed are increasing motivation (24%) and facilitating interaction (18%). These two advantages that are the most frequent in the review coincide with the findings of other studies about AR in education (Bacca et al., 2014; Diegmann et al., 2015; Chen et al., 2017; Fombona et al., 2017).

The third most frequently reported advantage refers to the fact that the AR catches the interest of students with disabilities or with special educational needs (SEN) (12%). This is considered an interesting finding because it is a key element when considering inclusive education. In this sense, several studies analyzed show the benefits of AR in working with students who have SEN, evidencing the work with the following populations: auditory limitation (Carvalhoand Manzini, 2017), visual limitation (Lin et al., 2016), autism (Tentori et al., 2015), attention deficit hyperactivity disorder (Lin et al., 2016b), dyslexia (Persefoni et al., 2016).

The fourth advantage identified is the low cost of implementing this technology in the classroom (8%). Although some vision devices are expensive, “AR provides tools for rapid and low-cost presence” (Zainuddin et al., 2010; Ab Aziz et al., 2012; Chen and Wang, 2015; Hsiao and Rashvand, 2015) and therefore it becomes a good tool to support processes in the classroom.

Helping with immediate memory has been identified as the fifth advantage most frequently stated in the studies, something that is also called short-term memory (6%) (Vullamparthi et al., 2013; Cihak et al., 2016; Martín-Sabarís, 2017).

The order of the rest of advantages is as follows: efficiency in the learning process (6%) (Fernandez et al., 2015; McMahon et al., 2015; Sytwu and Wang, 2016). Development of cognitive skills (4%) (Benda et al., 2015; Bülbül et al., 2016). The student-centered nature of technology (4%) (Tobar-Muñoz et al., 2014; Tentori et al., 2015). Reinforcement of student attention (4%) (Vinumol et al., 2013; Escobedo and Tentori, 2014). Enjoyment in the training process (4%) (Sheehy et al., 2014). The exploration and easy technological use by the student (4%) (Lucrecia et al., 2013; Marín Díaz, 2016). The satisfaction generated for the student (4%) (Chang et al., 2013; Sahin et al., 2018), and the most realistic perception provided (2%) (Miundy et al., 2017).

These findings show that AR is a technology that favors inclusive education.

Most of the studies do not mention AR limitations for inclusion (54%), which is a high percentage that leaves open the possibility of expanding the research to know in detail what are the limitations in using AR in inclusive education and other contexts.

Among the most important limitations we can mention would be the limited number of subjects in the sample size (22%). The fact that these studies indicate problems in expanding the sample in the research. According to this literature review, most studies have had <10 participants, which is considered to be a very small size (Zainuddin et al., 2010; McMahon et al., 2016; Cascales-Martínez, 2017). However, there are other issues that arise, for example, there are not many students in the same group or school institution with special educational needs and they are generally presented dispersed when applying the evaluation of the research. For this reason, these studies should be replicated in other similar populations (McMahon et al., 2015).

In order of relevance, the following limitation identified is the need to connect to the internet (5%), the fact that the application of AR, in some cases, requires good internet connectivity (McMahon et al., 2016). If that is not the case, this may hinder the application of AR technology.

Further limitations of the AR in supporting inclusive education identified with high frequency in the review are:

1. technical problems at the time of using the application (4%), which according to the authors is important when the research focuses on students with disabilities, either physical or mental, because in those cases the levels of frustration must be controlled very well. In this sense, the preparation and planning of the experiences with AR and what are the internet requirements must be rigorous (Sytwu and Wang, 2016).
2. lack of research on using mobile AR (MAR) in education with SEN (4%). Despite the fact that using mobile devices in the educational field is not a very recent technological innovation, more research is still required on using it with diverse groups, in inclusive education) (Sheehy et al., 2014).
3. qualified staff is required (4%); especially teachers (Colpani and Homem, 2016) and/or assistants are usually required to have basic digital knowledge for adopting ICT (Marín Díaz, 2016). However, for the creation of augmented content the level of complexity may increase.
4. it is not possible to replicate or repeat the study in another scenario (4%), as these are studies related to inclusion and special needs (SEN). They address problems of students with SEN, and therefore, these studies can hardly be repeated in other educational settings, although there may be exceptions (Chen and Wang, 2015).
5. the difficulty in recruiting participants for the study (4%), which explains the other limitation listed above, related to the size of the sample. Researchers report difficulty in finding students with SEN and acquiring the necessary documents to allow them to participate in the studies, including those needed from their parents (Lee et al., 2018).

Other limitations with less frequency in the studies are:

1. Luminosity difficulties (2%); luminosity is a requirement of AR technology, especially regarding markers, because good lighting must be available around markers.
2. AR applications do not allow adding 3D images in application mode (2%), meaning it is generally not possible for the user to change or add images.
3. Long-term results of the use of AR are needed to favor inclusion (2%). The use of AR in long-term educational inclusion should be analyzed and investigated in order to verify the time and the effects of this type learning for students.
4. Requires training in digital competence for students (2%). In some cases, students with SEN do not know how to use the technology needed (Cascales-Martínez, 2017), therefore, planning and prior training is recommended. This subcategory is related to the previous one, listed above, referring to qualified staff.
5. The novelty effect could produce bias in the results of the research (2%) (Cascales-Martínez, 2017). Which could be related to the student's fixation on technology and as a consequence, paying little attention to educational content. A good use of time and didactic methodologies are recommended in order to avoid distractions in this regard.
6. The use of only one tool to collect information (2%). Some studies used only one information collection tool (Zainuddin et al., 2010), usually surveys and/or interviews, however, in the case of children with different SEN it is recommendable to look for several sources of information collection in the same study.
7. More research is needed to prove acceptability in school environments (2%). In the case of inclusion, research should always be expanded in looking for alternatives when using AR so as to avoid excluding or marginalizing any group, not only SEN students, but also other populations at risk of marginalization.

About the types of devices used to favor educational inclusion through AR we have that the most outstanding are handheld devices (68%). Which is a finding that confirms that using AR applications on mobile devices supports educational inclusion, tablets and smartphones being the most widely used (Hsiao and Rashvand, 2015). PC and Web Cam (12%) come second, especially using them when caring for children with autism spectrum disorders (ASD) or intellectual disabilities which make using devices, such as desktop PCs necessary. Devices or screens mounted on the head occupy the third place (6%). These have been considered for cases of people with hearing and vision impairment, and autism (Fernandez et al., 2015; Sandnes and Eika, 2017; Sahin et al., 2018). Glasses (4%) are used to improve deaf students' communication in ordinary schools (Parton, 2017; Ioannou and Constantinou, 2018). Finally, large-screen projectors (2%), although very rare in the studies analyzed, have also been used for special cases in teaching primary school level mathematics (Cascales-Martínez, 2017).

On the category of educational field of application, taken as reference UNESCO, 2013 international standard classification 2013 we found that more than half of the studies analyzed are focused on elementary school students (58%) with different SEN. Lower secondary education ranks second in AR (12%). These two fields of application cover 70% of the studies, which is revealing, and it confirms the global situation of favoring inclusiveness mostly in primary education (Lindsay, 2018). In the third place is long-distance education (8%); here we can reference the study of Tesolin and Tsinakos (2018) which focused on developing three strategies for the elimination of systemic barriers in distance education in regard to inclusive education. Lastly, early childhood education, short cycle of tertiary education and undergraduate or equivalent studies are not reported in the literature reviewed; a possible explanation is the difficulty of having an adequate sample to carry out studies at these educational levels.

As the most relevant challenges, we can observe the long-term use of AR in different environments or contexts (10%). In this sense, the solutions found in trying to support inclusion make each study applicable to a specific context, but nevertheless, studies also indicate that solutions created to favor students with SEN can benefit all students (Meyer et al., 2014). Therefore, the use of solutions developed in varied contexts should be promoted and applied for a longer time in order to verify their effectiveness. Secondly, lowering costs in some technologies for AR vision (8%) becomes an important need according to the authors of the studies linked to the review. While many devices for AR are inexpensive, which was considered an advantage, there are other devices of a higher cost, such as different types of glasses. This is considered another challenge because educational institutions usually have a low budget to implement solutions that favor educational inclusion. Thirdly, there is the need to improve the hardware of handheld devices and their configuration potential (4%), so as to ensure that mobile devices offer quality audio and video and ensure an improved AR experience.

Other challenges that arise are: (1) creating personalized learning activities (2%); (2) allowing to change the settings, such as controlling the sound (2%); (3). User connection limitations with the Kinnect device (2%), a tool that is used in some cases as a motion sensor combined with AR to address some issues for children with disabilities although when the number of participants exceeds 6 it is not possible to use it anymore; (4) creating strategies to avoid distraction in students (2%); (5) enabling the use of AR in the learning processes of students with visual limitations (2%). Although there are reported studies on the use of AR in students with vision impairments, the possibilities of use with students who are totally blind are very limited (Marín Díaz, 2016). However, based on the contribution of Azuma et al. (2001) AR is not limited to the sense of sight, but also to others senses, such as hearing, smell and touch, generally used by students who are totally blind.

- F2: Different types of AR that are most promising in supporting inclusion.

When talking about inclusive education, researchers worked with markers mostly (84%), a result that is similar to that in other AR studies for education (Bacca et al., 2014), a reason for this being that they are stable and allow a better follow-up and efficiency. Markers are graphic symbols that contain patterns that are easily recognized by the software of AR, through any camera, which allows to trigger objects superimposed in 3D, generally (Wojciechowski and Cellary, 2013).

On the other hand, the second category in the table are those based on RA location (4%), something that requires the use of devices with accelerometer and compass as a main requirement and includes access to Internet with GPS. This type was especially used with students with intellectual disability and Down syndrome (Smith et al., 2017).

- F3: Types of research designs considered to evaluate the use of AR in inclusive education processes.

The following table shows the research methods that were used: “qualitative-exploratory case study” (22%), “qualitative-descriptive” (24%), and “mixed methods” (16%). These were the most used methods evidenced in the documents analyzed, the others mentioned below have been less applied: “literature reviews or studies case” (6%), “Single Subject Designs” (12%), “quasi-experimental design” (8%), “literature reviews or case studies” (6%), “pre-experimental design” (2%), “quasi-experimental design” (8%), “pure experimental design” (2%), “transversal research” (8%). Findings on single subject designs are consistent with previous studies (Horner et al., 2005; Gast, 2010) (Table 3).

**Table 3:** Research methods used to evaluate AR in inclusive education.

**Designs**	**(%)**
**QUALITATIVE DESIGNS**	
Qualitative-exploratory study	22
Qualitative-descriptive study	24
**ETHNOGRAPHIC RESEARCH**	
Literature reviews or case studies	6
**QUANTITATIVE DESIGNS**	
Single Subject Designs	12
Pre-experimental design	2
Quasi-experimental design	8
Pure experimental design	2
**MIXED METHODS**	
Quantitative + qualitative mixture	16
Transverse research	8

In relation to the samples, 48% of studies were found to have used samples of ten or fewer individuals, and 16% of the studies used between 11 and 30 participants. These results coincide with the limitation reported in the RQ1 on the difficulty to recruit participants. On the other hand, 10% of the studies report between 31 and 200 individuals. No studies with a sample size >200 (0%) were found, confirming the limitation exposed in RQ1. Finally, the methods of data collection used in the studies were questionnaires (24%), interviews (20%), case observation (12%), focus groups (10%), survey (8%), and case study (6%).

- F4: Types of population included in the learning scenarios supported by AR.

When speaking of inclusive education, the term is generally associated with learning scenarios where students with disabilities or special educational needs (SEN) participate (Ab Aziz et al., 2012). In the context of the review carried out, inclusive education is understood as offering learning scenarios supported by AR in order to generate opportunities for all students, taking into account individuals with disabilities and also excluded groups, such as ethnic minorities, immigrants, among others (Blanco, 2008).

Table 4 shows the population identified as target group in the studies reviewed, classifying them according to two categories: excluded groups and individuals with disabilities. With respect to the category of excluded groups, the largest number of studies were identified in the sub-category: Individual learning differences/different skills (16%). Only one study with ethnic minorities related to an indigenous population of Cauca in Colombia and their culture (2%) was identified in the review. Likewise, related to working with elderly populations, a study focused on training the elderly was identified (2%). No studies are recorded with the following target groups: victims of violence, religious minorities, immigrants, homeless people and/or population with exceptional talents. Working with these groups would be promising due to their great linguistic and cultural diversity. Researchers are encouraged to carry out research on the use of AR, since these groups have a high probability of being excluded from an educational system according to UNESCO. In particular, the exceptional talents of individuals can benefit from the use of AR methodologies or frameworks customized to their exceptional capabilities (Jolly and Hughes, 2015).

**Table 4:** Types of population in AR for inclusive education.

**Populations**	**(%)**
**EXCLUDED GROUPS**	
Individual differences in learning/different skills	16
Ethnic minorities	2
Senior citizens	2
Victims	0
Religious minorities	0
Immigrants	0
Homeless people	0
Exceptional talents	0
**BY DISABILITY**	
Physical or motor disability	4
Sensory disability	
Visual deficiencies	8
Hearing impairment (DHH)	20
Intellectual disability (ID)	14
**LEARNING DISABILITY**	
Language deficiencies	6
Dyslexia	2
Attention deficit hyperactive disorder (ADHD)	6
Characteristic deficiencies	2
**PSYCHOLOGICAL DISABILITY**	
Cognitive deficiencies	2
Autism Spectrum Disorder (ASD)	18
Down syndrome (DS)	2
Systematic reviews of literature	2

With respect to the Disability category, 20% of the studies were focused on individuals with hearing impairments (DHH), given that the AR allows the use of mobile devices and the visual channel is often preferred for perceiving information. The applications developed for this population combine videos with other visual tools or interactive multimedia (Parton et al., 2010), also promoting the use of glasses for AR and QR codes (Parton, 2017). On the other hand, 18% of the studies have also addressed the needs of individuals diagnosed with Autism Spectrum Disorder (ASD), since AR facilitates the creation of applications recognizing facial emotions, which represents a difficulty for individuals diagnosed with ASD (Chen et al., 2015). This helps teachers reduce their workload and supports better concentration and motivation in children with Autism (Escobedo and Tentori, 2014).

We found that 14% of the studies focus on individuals with intellectual disabilities. In this field AR has influenced the treatment due to its low cost and the use of gamification (Colpani and Homem, 2016). Vinumol et al. (2013)created an interactive textbook for children with learning disabilities due to neurobiological disorder, using AR, video and images to enrich learning by interacting with the exhibits. McMahon et al. (2015) conducted a study with students with intellectual disability and autism measuring their ability to independently make decisions to navigate. The purpose was to help them travel to unknown places in a city, and students did so with more success using AR, compared to Google Maps and a paper map. Lin et al. (2016) also explored the use of AR in children with different intellectual disabilities to facilitate the learning of primary elementary geometry. They found that AR can increase motivation and tolerate frustration in children with this type of special needs. In another case, for teaching science vocabulary in post-secondary education, McMahon et al. (2016) conclude that the students managed to acquire the proposed definition and knowledge.

Other sub-categories of populations that were less addressed in the studies according to the review carried out were: visual limitations (8%), language deficiencies (6%), attention deficit hyperactive disorder (ADHD) (6%), physical or motor disability (4%), characteristic deficiencies (4%), cognitive deficits (4%), dyslexia (2%), down syndrome (2%), among others.

The main results of the purpose of the studies analyzed in this review have been: their application in inclusive education (20%), “improving the level of understanding in students with intellectual disabilities” (16%), and “eliminating or diminishing the hearing barrier, improving communication” (14%).

Other results were: eliminating or reducing visual barriers (8%), increasing the recognition of emotional expressions (8%), eliminating or reducing reading problems (6%), facilitating access to information (4%), eliminating or reducing motor barriers (4%). Improving vocabulary issues for people with disabilities (4%), employment for students with intellectual disability (ID) and autism (4%). Improving the level of attention and behavior (2%), detecting movements (2%), reducing the workload of teachers (2%), attending to student diversity (2%), improving navigation or displacement in students with disabilities (2%), conservation of cultural features (2%). All were focused and centered on inclusivity.

- F5: Frameworks or models for attention to diversity used to support the creation of AR applications that facilitate processes of educational inclusion.

Universal Design for Learning was identified as the most widely used framework in studies that support inclusive learning (12%). The reason is that this framework has been widely approved for inclusive education work around the world. Other frameworks identified were: “AR BACA Sind” (2%), an AR framework aimed at students with Down syndrome. “AR gamification” (4%) which proposes to help the learning process of children with intellectual disability in general and “FlarManager” (2%), an AR framework for Flash, used for the development of applications of students who are deaf. Another example is “co-CREARGBL” (2%), a learning methodology based on games which proposes three stages (training, iterative design and Class Evaluation). In this case, for the validation process the authors designed an application oriented to learning and increasing motivation in an indigenous community of Southwest Colombia. In total, five frames were found.

Although the previously mentioned frameworks were identified, 78% of the studies do not refer to the framework that was used to meet the diverse needs of the students. This shows that more research is needed in order to establish what are the theoretical bases of the solutions designed for attending diversity with the help of AR and also, how effective they are in achieving real educational inclusion.

Table 5 shows the applications for inclusive education: “Mobis,” “Tabletop system,” “SAM,” “AR-SiD,” “IVRALS,” “KanHan,” “Paint-cAR,” “Cuetaya,” “Gremlings in my mirror,” “ARCoach,” “Troyoculus.” In total, 11 reported applications. It is striking that 76% of the records do not mention the development of any application to attend these individuals, however, one reason may be the use of authoring tools, such as “Aurasma” or “Layar,” where it is possible to create and apply AR online easily.

**Table 5:** AR applications for inclusive education.

**Applications**	**(%)**
Mobis	4
Tabletop System	2
SAM	2
AR-SiD	2
IVRARLS	2
KanHan	2
Paint-cAR	2
Cuetaya: Land of Colors	2
Gremlings in my mirror (Game)	2
ARCoach	2
Troyoculus	2
Does not register full application	76

Since there are different types of disability and of population at risk of exclusion in education, it is necessary to investigate and create more applications to meet these special needs.

- F6: Types of technology, including assistive ones, developed to support using AR for educational inclusion.

Technologies used with AR in inclusive education are mainly mobile AR (MAR) (44%). This result is easily explainable, it coincides with findings for RQ1, the use of handheld devices and with findings for RQ2, AR based on markers, which are the most used in inclusive contexts. The “based on vision” significance (16%) is another result that can be explained and confirms findings for RQ4, related to the population attended the most, groups with hearing deficiencies, through multimedia tools with AR.

The next subcategory in importance, based on sensors (6%), refers to those sensors used to search and record the movements of students with some physical or motor disability. Kinnect devices (6%) also help to control movements in combination with AR. Next, 3D (6%), may have been included by more studies in their applications, but only 3 studies mentioned it. The following entries are: “TextBook or Storybook” (4%), “ARVMS” (2%), “Oculus Rift (helmet)” (2%), “Tesseract OCR” (2%), “Face detection module” (2%), “ARCM” (2%), “SixthSense” (2%).

- F7: Platforms and authoring tools considering the diverse needs of users in the process of creating AR-based learning experiences.

Table 6 reports the platforms and software used to develop the applications of AR; considering that some studies used more than one tool, a classification of the category has been made as it follows. “SDK's or libraries”: “Vuforia” (8%), “ARToolkit” (6%), “FLARToolkit” (2%), “Vidinoti” (2%), “NyARToolkit” (2%), “ARTag” (0%), “ARToolkit for Unity” (0%). A possible explanation for the use of Vuforia is the fact that it is integrated into the new versions of Unity and has a free and commercial version and is “stable and efficient and offers several features, which allows the capacity of mobile applications and frees the developers of the technical limitations” (Amin and Govilkar, 2015).

**Table 6:** Software tools used with AR for inclusive education.

**Softwares**	**(%)**
**SDK'S**	
Vuforia	8
ARToolkit	6
FLARToolkit	2
Vidinoti	2
NyARToolkit	2
ARTag	0
ARToolkit for Unity	0
**PROGRAMMING AND DEVELOPING LANGUAGES**	
Scratch	4
Visual Studio	4
C #	2
UnityAR	2
Java o JSP	2
Flash	2
**AUTHORING TOOLS**	
Aurasma (HP Reveal)	10
Layar	4
AuthorAr	2
Wikitude	2
**SOFTWARE FOR 3D MODELING**	
Blender	4
Irrlicht3D, OGRE3D	2
**OTHERS**	
The Joiner algorithm, AR and AVSR	2
Not mentioned	52

In the next category, “programming and developing languages” the following are reported: “Scratch” (4%), “Visual Studio” (4%), “C #” (2%), “UnityAR” (2%), “Java or JSP” (2%), “Flash” (2%). In this group the results don't make for big differences between them.

The registered authoring tools were: “Aurasma (HP Reveal)” (10%), “Layar” (4%), “AuthorAR” (2%), “Wikitude” (2%). The possible explanation is that Aurasma now HP Reveal, is one of the most used tools for browser in the world, easy to use, available for Android and iOS and free (Delello et al., 2015).

Regarding software for 3D modeling, the following were reported: “Blender” (4%), “Irrlicht3D, OGRE3D” (2%). “Blender is a multiplatform computer program, dedicated especially to modeling, lighting, rendering, animation and creation of three-dimensional graphics, it is a free software” (Rosales, 2015); in recent years this system has gained more followers (Dovramadjiev, 2015).

In the category “Others,” only “The Joiner Algorithm” (2%) appears, and 52% of studies, more than half, curiously, do not mention the type of software tool used to create AR applications. More research is needed in order find more data and expand this information.

- F8: Effects of AR experiences identified in this systematic review.

Below, in Table 7, are the registered and related effects, and the first one refers to improving communication in students with disabilities (24%). If we compared this result with findings for RQ4 in the disability category, where the highest percentage of studies had to do with “auditory deficiencies,” which in summary improves communication, we'll understand why this is the first entry, because it represents a fundamental reason for the effect analyzed here. The second entry, “it raises interest, attention, motivation and school performance in students with SEN” (22%), can be explained by findings in literature. Various studies have contributed to these effects in education (Liu and Chu, 2010; Di Serio et al., 2013; Bacca et al., 2014; Chang et al., 2014; Akçayir and Akçayir, 2017), and the same can be said for children with special educational needs. Next, “the teacher can create personalized content for the child” (8%), refers to how experiences gave way to this possibility, something that increased the opportunities of an inclusive and personalized education. The entry: “increase of knowledge of the subject in students with SEN” (8%) has to do with results of different evaluations that highlighted the fact that students with different disabilities managed to acquire the knowledge proposed in the specific topic (Cihak et al., 2016; McMahon et al., 2016; Cascales-Martínez, 2017). Then, “improve the teaching of work and employment skills” (6%) has to do with three documents that were inclined toward non-traditional teaching in people with disabilities who aimed to improve some skills needed to bring these individuals closer some type of employment. The entry: “motivates physical activity in students with disabilities” (4%) has to do with work aimed at children with mental problems (Lin et al., 2016a,b). The following: “to improve navigation through digital maps” (4%) refers to studies trying to make students able to move geographically on their own. The next entries are: “increases access to distance education” (2%), and “the burden of the teacher with the disabled child goes down” (2%), which has to do with the fact that, in some cases the teachers attending these types of students do not have the support needed or there are not enough teachers, and AR tools aim to reduce this burden in teaching (Escobedo and Tentori, 2014). The following entry is “to improve the physical and mental health of the elderly” (2%) and the next one is “improves knowledge of indigenous culture and traditions” (2%); however, only one study was focused on preserving indigenous traditions (Pinto et al., 2017), also considered herein as excluded groups.

**Table 7:** AR effects on inclusive education.

**Effects**	**(%)**
Improves communication in students with disabilities	24
Raises interest, attention, motivation and school performance in students with SEN	22
Does not mention	16
The teacher can create personalized content for the child	8
Increases knowledge of the subject in students with SEN	8
Improves the teaching of work and employment skills	6
Motivates physical activity in students with disabilities	4
Improves navigation through digital maps	4
Increases access to distance education	2
Reduces the burden of teachers of students with disabilities	2
Improves the physical and mental health of the elderly	2
Improves knowledge of indigenous culture and traditions	2

In general, all studies ended with a minimum of positive effects on students with different needs, and, depending on the case, it improved their experience in the educational system taking advantage of the benefits of the AR.

## Discussion and Challenges

The first challenge regarding the use of AR in the educational field and in particular in favoring processes of educational inclusion, refers to the educational levels linked to current studies. More research is needed in: early childhood education and the short cycle of tertiary education and degree or equivalent, all this taking into account the international standard classification of education (ISCED).

Another important future issue to consider is directing research toward educational fields where AR studies were not reported, such as engineering, agriculture, forestry, business, fishing, veterinary, among others, considering the diverse needs of students.

A third challenge has to do with the use of different types of AR to support educational inclusion. It is aimed at exploring the use of markerless AR, or AR without a marker. By taking advantage of the best technological conditions of current mobile devices, such as cameras and sensors (given that the most studies reported the use of markers and QR codes). Not needing markers could generate greater ease of use for students.

The fourth challenge is oriented toward the methods of data collection, going beyond questionnaires and interviews, which were reported as the most used methods. It is proposed to include other methods of data collection directly in the inclusive classroom, in order to avoid unreliable evaluations or giving desirable responses in the research (Castells, 2016). Observation and case studies can help to a greater depth in the analysis of the investigation.

The fifth challenge is to further diversify the population served; most of the studies analyzed tended to address the educational needs of populations with hearing impairment and autism. It is necessary to expand and diversify the research to cover other needs, such as dyslexia, Down syndrome, attention deficit, hyperactivity, among others. Likewise, future research should include vulnerable groups such as: migrants, ethnic minorities, exceptional talents, which have not been addressed in research on the use of AR in education or have not been reported so far.

The sixth challenge identified is the need to expand research in developing models, frameworks and methodologies that use AR to favor educational inclusion, considering different populations that benefit from the relationship between technology and pedagogy. The development of these models, frameworks and methodologies should be done by multidisciplinary working groups that would not only focus on the technical characteristics, but have a strong didactic and psychological approach as well. We only found five frameworks oriented to a specific need of a disability, without considering wider contexts of diversity. No models and methodologies were reported. In this sense, frameworks supported by multimodal learning and AR (Gilakjani et al., 2011) can be successful due to the multiplicity of channels and means of communication where the learning process is directed, guiding the creation of learning scenarios in contexts of diversity. Likewise, the consideration of the universal design for learning, a conceptual framework for attention to diversity that has been widely validated, could provide inputs from the conceptual standpoint of these models, frameworks and methodologies.

The seventh challenge is defining truly diverse samples, not oriented to isolated populations. Most of the studies analyzed state that they are oriented to specific types of people with disabilities, such as those who are deaf, blind, have Down syndrome, etc. Only a very small number of studies (3) define truly diverse samples.

The eighth identified challenge is the need to create and put into operation platforms or authoring tools that make use of AR and that are directly oriented to the attention of the diverse educational needs of students at different educational levels.

The ninth and last identified challenge refers to the evaluation of effects on medium and long term on the use of AR in different contexts; this would verify the real impact of this type of technology in learning scenarios that welcome diversity. Generally, the studies analyzed report a short-term evaluation through experimental studies carried out at a specific time. It is recommended to carry out experimental studies that allow verifying the impact of the use of AR over time.

## Conclusions

A systematic review of literature on the topic of AR was carried out and applied in the context of educational inclusion; a total of 50 studies were analyzed, conference articles, book chapters and journal articles, applying the method of content analysis. The following factors were considered in the selected studies: field of education, advantages, limitations, uses, challenges and educational scope. In addition, we considered the purpose of the studies and research methods used, as well as the type of sample, and the population its effects.

Here is a brief summary of the main conclusions:

- The number of studies published on AR for educational inclusion between 2013 and 2018 has been maintained at an average of eight per year, with 2015 being the year when most articles were published, an average of 11.
- In the field of education sciences is where most studies have been applied, the least explored are in engineering, manufacturing and construction.
- Among the advantages reported were the motivation, interaction and catching the interest of the student with disability, all criteria that help inclusive education.
- The main limitations are small samples (often only a single subject is included) or the need for internet connectivity, considering this service is deficient or does not exist for certain populations.
- The most used devices are mobile or handheld devices, followed by the desktop computer or PC.
- Among the most reported challenges would be long-term focus and use in other environments or contexts. Most studies have not considered extending the time for testing and evaluating, nor extending to other scenarios outside those used initially.
- A high percentage of studies were applied to primary education, but education for secondary school, early childhood and short-term education should be explored in the future.
- The type of AR used most is based on markers, then geolocation; studies have not yet been explored without markers.
- Most studies used small samples, ten or less individuals, and some included between 11 and more participants.
- Information collection was mostly done through questionnaires and interviews.
- Regarding the population served, the studies were generally inclined toward students with disabilities, leaving aside another population or groups that are also excluded from the education system, something that could be explored in the future.
- Few frameworks for inclusive education were reported, despite the existence of various problems to be addressed.
- Some technologies have been used and combined in order to apply them in inclusive education, most of them in mobile devices, expanding the use of glasses and sensors.
- Vuforia is the library most used in the creation of AR applications, and authoring tools are Aurasma and Layar; however, it should be noted that most don't mention this aspect in their studies.
- The greatest effect is improving communication skills in students with disabilities, especially oriented to those with hearing problems, where more work has been done and therefore there are more studies on this topic.

The present work contributes to expanding the current state of research in the field of the application of AR in inclusive education, covering not only aspects of disabilities, but of other groups possibly excluded from the educational process, aiming at identifying the benefits and effects to be considered by future studies.

## Data Availability

All datasets generated for this study are included in the manuscript and/or the supplementary files.

## Author Contributions

JQ, SB, and RR: conceptualization. JQ and SB: formal analysis, investigation. JQ, SB, and RR: methodology. JQ, SB, RR, and GV: writing—original draft. JQ, SB, RR, and JC: writing—review and editing.

### Conflict of Interest Statement

The authors declare that the research was conducted in the absence of any commercial or financial relationships that could be construed as a potential conflict of interest.
